# Deducing fast electron density changes in randomly orientated uncrystallized biomolecules in a pump–probe experiment

**DOI:** 10.1098/rstb.2013.0332

**Published:** 2014-07-17

**Authors:** K. Pande, P. Schwander, M. Schmidt, D. K. Saldin

**Affiliations:** Department of Physics, University of Wisconsin-Milwaukee, Milwaukee, WI 53211, USA

**Keywords:** X-ray free-electron laser, proteins, time-resolved

## Abstract

We propose a method for deducing time-resolved structural changes in uncrystallized biomolecules in solution. The method relies on measuring the angular correlations of the intensities, when averaged over a large number of diffraction patterns from randomly oriented biomolecules in solution in a liquid solvent. The experiment is somewhat like a pump–probe version of an experiment on small angle X-ray scattering, except that the data expected by the algorithm are not just the radial variation of the averaged intensities. The differences of these correlation functions as measured from a photoexcited and dark structure enable the direct calculation of the difference electron density with a knowledge of only the dark structure. We exploit a linear relation we derive between the difference in these correlation functions and the difference electron density, applicable for small structural changes.

## 1. Introduction

The method of time-resolved crystallography [1] has given us insights into rapid variations of the structure of biomolecules via pump–probe experiments involving photoexcitation by a laser followed very quickly by measurement of X-ray diffraction patterns. Changes in the structure are then found by established methods [2,3]. We present here computer simulations that suggest that such structural changes may be determined even from an ensemble of biomolecules in solution.

The X-ray free-electron laser (XFEL) is a new instrument that promises to revolutionize our study of the atomic architecture of matter [4,5]. The brightness of the X-rays produced by this instrument is some 10 billion times greater than any existing X-ray source (including present-day synchrotrons). This allows the possibility of measuring signals from scattered X-rays of large single molecules, such as proteins. The traditional limitation of X-ray flux for fragile biomolecules can be circumvented completely due to the fact that this very bright radiation is delivered in ultra-short pulses [6–8]. Although the molecules under study will undoubtedly suffer catastrophic radiation damage, the shortness of the pulse enables a signal (albeit weak) to be measured from the particle before its disintegration [4]. In common with other theories developed for extracting structural information from such molecules, we will first assume [9] that a single molecule is hit by the X-ray beam at a time, with random orientations each time. The fact that the particles are hit by the incident X-ray pulses in random orientations enables the full three-dimensional structure of the scattering object to be found from the ensemble of diffraction patterns without deliberate rotations of the object with a goniometer, as is usually done in protein crystallography. However, despite the huge increase in the brightness of an XFEL beam over all previous X-ray sources (a factor of about 10^10^), the reduction in size from even a 0.1 mm diameter crystal commonly used in X-ray crystallography, to a single molecule (typically of diameter 10^–8^ m) is even greater (typically a factor of approx. 10^−12^). Consequently, very few X-ray photons are scattered by a single molecule. Nevertheless, the other feature of XFEL radiation, namely that it is delivered in pulses of a few femtoseconds duration allow a recording of a large number of diffraction patterns per second, thus allowing the measurement of even millions of diffraction patterns from random particle orientations over the course of a single experiment. The search has therefore been on for the development of algorithms that can overcome the low signal-to-noise ratio of any single diffraction pattern by judiciously combining information from a large number of such diffraction patterns. Such an algorithm would allow structure determination of reproducible biomolecules by essentially an unlimited X-ray flux (because the scattering is expected to take place before the molecule's disintegration). This also would be, in one sense, a complete solution of the radiation damage problem. We suggest in this paper that it should also be possible to combine the ultra-brightness [10] of the radiation with the ultra-shortness of its duration [11] to enable the gathering of information never before possible, for example the changes in the structure of an uncrystallized biomolecule in solution as a result of some stimulus, such as photoexcitation, as a function of time since the photoexcitation. Because this time can be very short, the possibility then exists of experimentally following the course of rapid chemical reactions of such uncrystallized biomolecules, as is already done with crystallized ones [11] by the technique of time-resolved diffraction [1,12,13]. This may allow for the first time the study of biochemical reactions of molecules in aqueous solution in which they occur in nature.

An idea proposed for sample delivery of hydrated molecules to an XFEL beam is to inject a continuous stream of a solution containing the molecules into the sample chamber [14–16]. The incident X-rays then scatter off the protein solution. Initially, we assume a single particle per diffraction pattern, as do all other theories for the extraction of structural information from XFEL patterns, but in §6, we point out how this restriction may be relaxed.

## 2. Structural information from disordered ensembles of molecules

Due to the fact that the particles are presented to the XFEL beam in random orientations, the individual *intensities* will not be obviously related to intensities on other diffraction patterns. However, it has been pointed out [9,17] that the average angular correlations over a large number of such diffraction patterns can yield a quantity that is directly related to the three-dimensional diffraction volume of a single particle. If an oversampled version of the three-dimensional diffraction volume can be extracted from this ensemble of diffraction patterns, modern iterative phasing algorithms [18,19] would be expected to be able to reconstruct the three-dimensional electron density of an individual particle from the multiple diffraction patterns expected to be measured in such an experiment.

**Figure 1.**
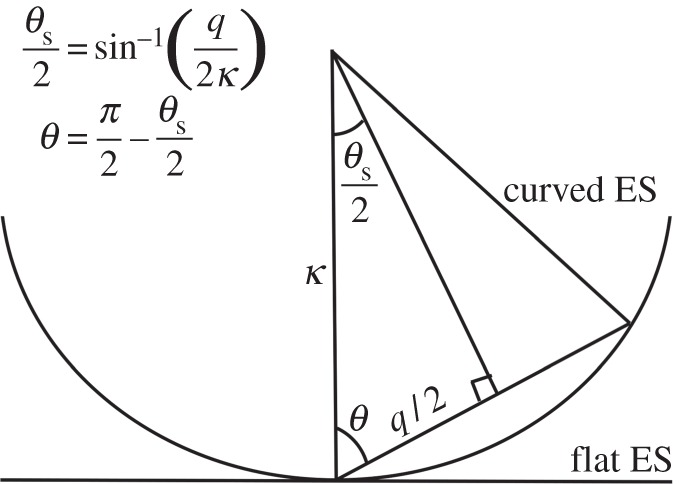
Schematic of diffraction geometry demonstrating the relationship between polar angle *θ*(*q*), scattering angle *θ*_s_(*q*), magnitude of scattering vector *q* and wavenumber *κ* = 2*π*/*λ*. ES, Ewald sphere.

We begin by defining an average over a set of diffraction patterns of angular pair correlation function between the intensities of a couple of resolution rings *q* and *q’* of diffraction patterns by2.1
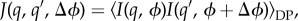
where 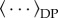 represents an average over all the diffraction patterns, DP. Note that the orientational averaging of the particles implied by the reasonable assumption that all molecular orientations are equally likely suggests that the LHS of the equation (2.1) will be independent of the value of *ϕ* chosen on the RHS. In this case, it is possible to show [9] that2.2
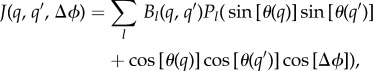
where2.3
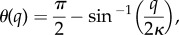
and *κ* is the wavenumber of the incident radiation. The angle *θ*(*q*) takes account of the curvature of the Ewald sphere, and is almost equal to *π*/2 radians for a small resolution ring or a flat Ewald sphere (figure 1). However, this formalism also allows one to take account of the curvature of the Ewald sphere very naturally. In this expression, also2.4
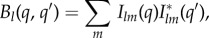
where *I_lm_*(*q*) are a set of spherical harmonic expansion coefficients of the three-dimensional diffraction volume of a single particle, and *P_l_* is a Legendre function. Finding the coefficients *I_lm_*(*q*) enables the reconstruction of an *oversampled* three-dimensional distribution of scattered *intensities*. Recent developments of iterative phasing algorithms [18,19] suggest that this is all that is needed to reconstruct the electron density of the particle giving rise to the scattered intensity distribution.

Although the quantities *B_l_*(*q*, *q’*) may be found quite straightforwardly by inverting equation (2.2), finding the *I_lm_*(*q*) coefficients in general from these quantities is far from easy [20], although where the particle has a known symmetry, for example, in the case of an icosahedral [21] or helical virus [22] this may be possible by exploitation of the known symmetry.

Another circumstance in which structural solution is possible is when what is sought is a small change in the structure from a known one. There are no symmetry restrictions to such a difference structure, only that it be close to the known dark structure (the same assumption as made in the difference Fourier method of time-resolved crystallography). Such a solution is of great significance in that it suggests the possibility of following the fast time variation of the structure of a photoexcited biomolecule of uncrystallized biomolecules in solution under conditions which somewhat mimic their functional states in living organisms. Any steric hindrance caused by neighbouring molecules in a tightly packed crystalline state is circumvented by not having to crystallize the molecules at all, which may also have experimental advantages. Such an experiment truly exploits not only the extreme brightness, but also the fast time structure of XFEL pulses.

We demonstrate this in the case of photoactive yellow protein (PYP), where earlier time-resolved studies have established that, 2 ms after photoexcitation, the primary change in the structure may be regarded as a *cis–trans* isomerization of its chromophore about its C2–C3 axis, and a change of the *χ*_3_ torsional angle of the side chain of the ARG 52 residue, to make room for the structural changes owing to the chromophore isomerization.

## 3. Proposed time-resolved experiment on dissolved molecules in random orientations

**Figure 2.**
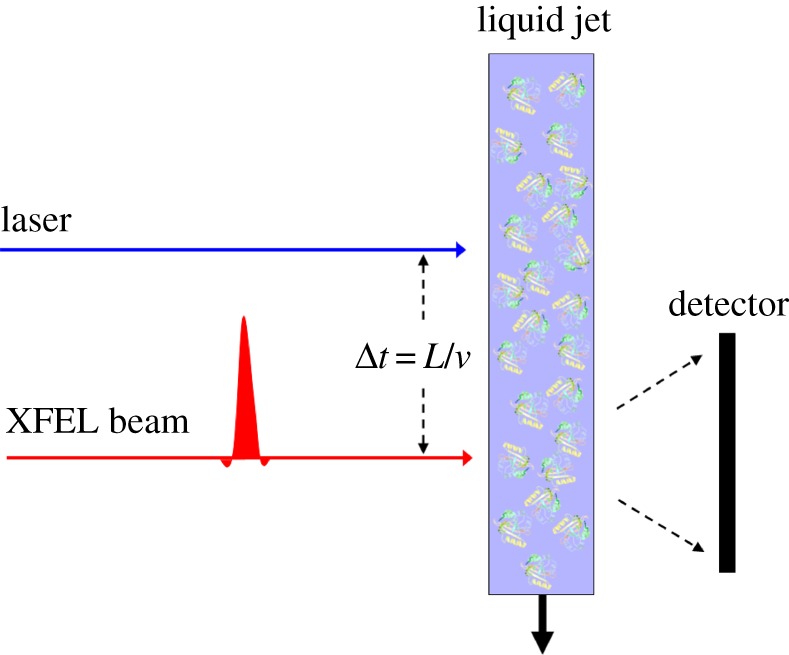
Schematic diagram of the proposed pump–probe experiment. A continuous stream of a solution of identical molecules in random orientations is introduced to the path of the X-ray beam after photoexcitation by a laser a controllable time before interrogation by the X-rays. The time delay Δ*t* between photoexcitation and X-ray incidence is controlled by varying the distance *L* between the positions of photoexcitation and X-ray incidence.

Imagine an experiment where a continuous stream of a solution of photoexcitable molecules is injected into an XFEL sample chamber in the usual manner of the so-called diffract and destroy experiments [4], but where a short distance *L* before the intersection of the X-rays from the XFEL, the molecules are photoexcited by a powerful laser. If *v* is the speed of the solvent stream (typically 10 m s^−1^), then the molecules will be illuminated by the laser a time Δ*t* = *L*/*v* before it is interrogated by the X-ray beam. Because the time Δ*t* is controllable by varying the distance *L*, this pump–probe experiment allows exquisite control of the time delay after photoexcitation. It is envisaged that a large number of diffraction patterns may be measured from different regions of the continuous solvent for a given time delay. Diffraction effects owing to solvent scattering may be removed by a Babinet principle argument also used in the theory of small angle X-ray scattering (SAXS) [23]. It is expected that structural information of the photoexcited molecules in the solution resides in the ensemble of such difference patterns [24] (figure 2).

## 4. Extraction of time-resolved structural changes from randomly oriented molecules

In our earlier paper [24], we demonstrated that the structural content of the *B_l_*(*q*, *q’*) quantities is extractable from a set of XFEL diffraction patterns of multiple randomly oriented biomolecules. For the case of an assumed structural change of a PYP molecule about 2 ms after photoexcitation which consists of a *cis–trans* isomerization of a chromophore and a simultaneous torsional angle rotation of the side chain of a nearby arginine residue, we showed that the presumed photoexcited structure can be found by optimizing the *B_l_*(*q*, *q’*) quantity of the photoexcited structure, starting at the value for the known dark structure and assuming that its change on photoexcitation may be characterized entirely by the variation of two torsional angles. Because the number of parameters varied in this case was just two, our simulations suggested the possibility of finding the correct photoexcited structure by performing an exhaustive search through the two-dimensional parameter space to minimize a reliability (or *R*-) factor between a simulation of the expected 2 ms structure and simulations of model structures for valid values of the structural parameters. Of course for a larger possible number of varied structural parameters, a more sophisticated search algorithm, such as simulated annealing [25], would need to be used. However, if absolutely nothing was known in advance of the photoexcited structural change, then such a simulated annealing method may not be practical, as the number of structural parameters to be varied may exceed the practical capability of such an algorithm [26].

In such a circumstance, an algorithm akin to the difference Fourier method of traditional time-resolved crystallography, which reveals the difference electron density of the entire molecule, from the experimental data alone, and a knowledge of only the dark structure would be desirable. We show below how such a difference electron density may be recovered from experimentally measurable quantities even from uncrystallized biomolecules in solution, with a knowledge only of the dark structure, just as in traditional time-resolved crystallography.

The idea is that, for small structural changes, one may take the variation of both sides of (2.4) to give4.1
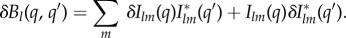


On the RHS, we interpret *I_lm_*(*q*) as the magnitude of the spherical harmonic expansion coefficient of resolution shell *q* of the (usually known) dark structure and *δI_lm_*(*q*) the corresponding quantity associated with the difference in the scattered intensity of the photoexcited and dark structures of a molecule. That is, one may write4.2
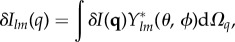
where **q** ≡ (*q*, *θ*, *ϕ*), d*Ω_q_* represents integration over the angles *θ* and *ϕ*, and4.3
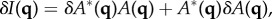
where *A*(**q**) may be regarded as the scattered amplitude from the dark structure and *δA*(**q**) that from the difference structure. Consequently, we can write4.4
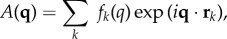
where *f_k_* is the form factor of an atom at position **r***_k_*, in the dark (unphotoexcited) molecule, and4.5
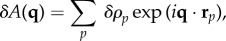
and *δρ*_*p*_ is the difference electron density at voxel *p* at position **r***_p_*. Substituting (4.4) and (4.5) into (4.3), we can write4.6
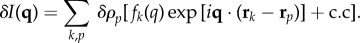
from which we deduce that4.7
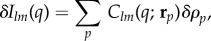
where4.8
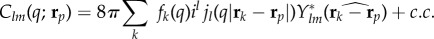
The quantity *j* in (4.8) is a spherical Bessel function, and *Y* is a spherical harmonic.

Substituting (4.7) and (4.8) into (4.1), we deduce that4.9
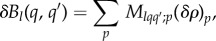
where4.10
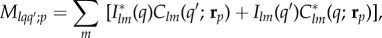
where all quantities on the RHS are known (and real) if the dark structure is known (as it usually is in time-resolved experiments) from which we deduce that4.11
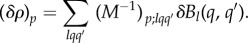
Note that because of Friedel's law, namely that *I*(**q**) = *I*(−**q**), the only non-zero values of *I*_lm_ and hence of the elements of *M* are those of even values of *l*. It should also be noted that4.12
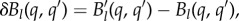
where *B’* is a quantity measurable from pump–probe experiments of the photoexcited structure, and *B* that of the dark structure. Thus, the RHS of equation (4.11) consists of a product of a column vector consisting of quantities *δB_l_*(*q*, *q’*) measurable by experiment, and a matrix *M^−^*^1^ which depends only on the known dark structure. This yields the sought quantity *δρ*_*p*_, which is the difference electron density at voxel *p* in the frame of reference of the dark structure, and is therefore easily superimposable on models of the molecule, as we see below. Unlike proposed methods of *ab initio* structure determination of viruses from angular correlations of XFEL intensities [21,22], the method we propose here does not depend on a knowledge of the symmetry of the sought difference structure, only that the difference electron density be small compared with the electron density of the dark structure (the usual assumption of time-resolved crystallography).

## 5. Model calculations

PYP is a popular (15 kDa) protein for time-resolved structural studies. To make a connection with previous work in time-resolved crystallography [27–30], we illustrate our method on the same molecule. As an initial test of these ideas, we simulated diffraction patterns from individual PYP molecules in random orientations. Of course, owing to a lack of long-range order, such diffraction patterns would be expected to be completely diffuse, with no Bragg spots. First, a set of 10 000 diffraction patterns from PYP molecules in a so-called dark state before photoexcitation were simulated, and then similar sets of 10 000 diffraction patterns each from various assumed excited states involving substantial structural changes to one or two residues. The dark-state structure was taken to be that deposited in the protein data bank (PDB) under the entry 2PHY. Various test structures were determined by making torsional angle rotations of various side chains. The calculations were performed on about 200 computer cores on the AVI computer cluster maintained by the University of Wisconsin-Milwaukee. Thus, for each set of 10 000 diffraction patterns, each core computed just 50 diffraction patterns from molecules oriented over a uniform set of orientations in SO(3), in order to simulate the expected range of orientations in which such molecules are likely to be presented to the X-rays in an XFEL ‘diffract-and-destroy’ experiment.

The angular correlations *J*(*q*, *q’*; Δ*ϕ*) were then calculated from each simulated diffraction pattern by the formula5.1
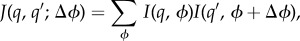
where *I*(*q*, *ϕ*) is the expected intensity on resolution ring *q* and azimuthal angle *ϕ* of each diffraction pattern. An average was then taken of the quantities *J* over all the simulated diffraction patterns following equation (2.1). From these quantities, the quantities *B’_l_*(*q*, *q’*) and *B_l_*(*q*, *q’*) for the photoexcited and ‘dark’ structures were calculated by inverting equation (2.2). The difference, *δB_l_*(*q*, *q’*) (4.1) between these two quantities, easily extractable from two sets of measured XFEL diffraction patterns, one from a photoexcited molecule and one from a molecule in a dark state, was then the basic simulated experimental data for our algorithm for recovering the difference electron density (*δρ*)*_p_* at voxel *p* in the frame of reference of the dark structure (see e.g. equation (4.11)) from sets of randomly oriented photoexcited and dark molecules of PYP. For the known dark structure, we evaluated the elements of the *M* matrix that also appears on the RHS, and took its inverse. The result of multiplying the two quantities on the RHS is the recovered difference electron density in the frame of reference of the assumed dark structure.

**Figure 3.**
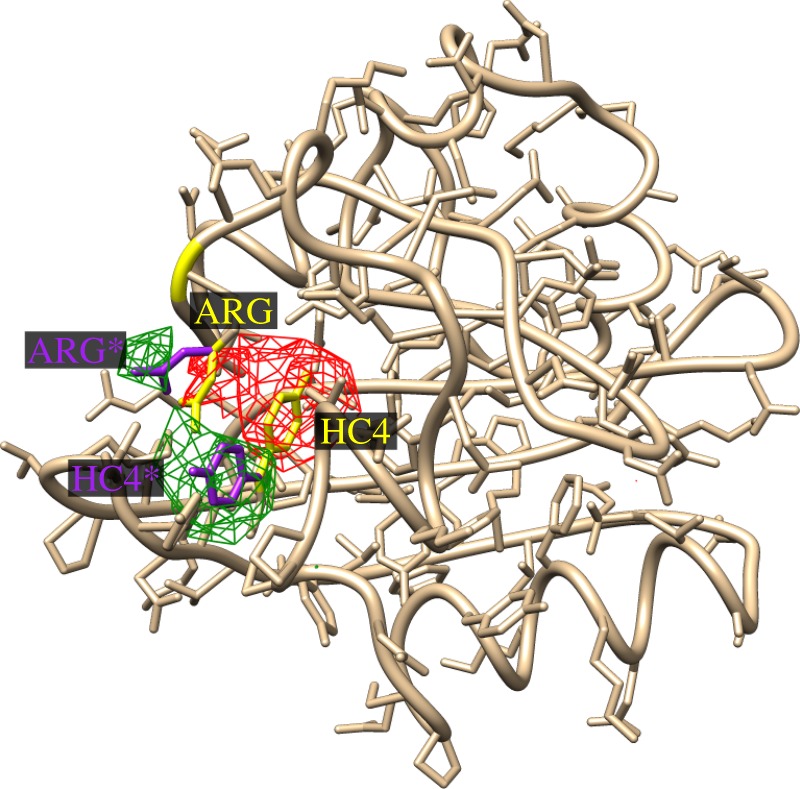
Difference map between the models of the photoexcited and dark structures of PYP assumed in the calculations. The difference between the two structures is that the chromophore (HC4 300) undergoes a *cis–trans* isomerization which causes it to swing outward towards the edge of the molecule, whereas the ARG 52 residue also swings outwards to make room for the new position of the chromophore in the photoexcited structure. Note that the red lobes of negative electron density are associated with positions of these residues (or functional groups) in the dark structure (yellow bonds) and the green regions of positive difference electron density with their positions (purple bonds) in the excited structure (although the red lobe on the original position of ARG 52 is somewhat cancelled by the fact that it lies between the green lobes of ARG 52 and the chromophore.

The difference electron density we so recovered for a hypothetical photoexcited structure owing to the assumed differences between the 2 ms and dark structures (consisting of a *cis–trans* isomerization of the chromophore which is a result of a 180° torsional rotation of the head of the chromophore about its C2–C3 bond, as well as a 77° change of the *χ*_3_ torsional angle of the side chain of the ARG 52 residue of PYP [27–30]), as shown in figure 3.

**Figure 4.**
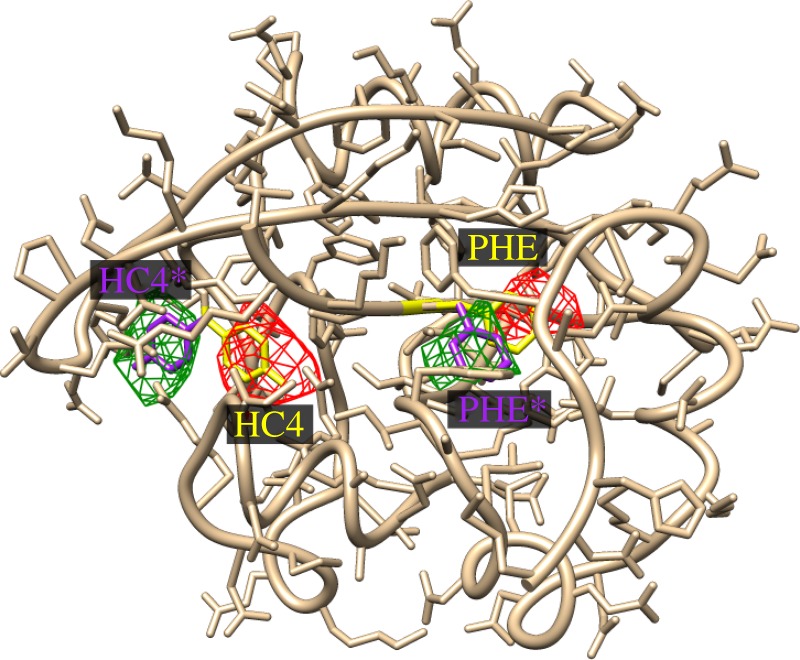
Recovered difference electron density from simultaneous torsional angle rotations in a hypothetical structure with structural changes in the widely separated chromophore and PHE 121 residues. Colour conventions same as figure 3.

Because, in this case, the two functional groups that change their positions, namely the chromophore and the ARG 52 residue are in close vicinity, the difference densities from the two are somewhat overlapping. In order to see what would be expected of changes in widely separated functional groups, we also tested a different hypothetical structure involving simultaneous torsional angle rotations of the chromophore, and the PHE 121 residue, as illustrated in figure 4. In this case, the difference electron density owing to the chromophore and the PHE 121 residue are widely enough separated for these difference electron densities to be clearly distinguishable.

## 6. Scattering by multiple particles per diffraction pattern

Next, we wish to comment on a feature of our algorithm which seems to be unique among theories proposed to date for the structure solution of biomolecules from XFEL experiments, namely that it seems to work equally well even if an ensemble of biomolecules in random orientations and random positions contributes to each diffraction pattern, as was realized by Kam [17]. This is only to be expected of a method that seeks averaged correlations over a large number of different particle orientations because, regardless of the relative particle orientations on a particular snapshot, the averaged values of these quantities over all particle orientations would be expected to be the same.

The importance of this for proposed experiments on individual protein molecules with a liquid jet [15] is that it would enable the use of a higher concentration of protein molecules than would be possible in an arrangement that illuminates just a single molecule. This increases the relative magnitude of the signal from the molecules compared with solvent and enables the extraction of structural information about a single particle from multiple identical particles in random orientations. Consequently, this would allow the possibility of structure determination of many biomolecules directly from conditions close to their operating conditions in nature, namely in dissolved aqueous environments, without the need for crystallization.

As mentioned above, in order to maximize the protein scattering from such a solution stream (and to minimize scattering by the aqueous solution), it would be best to use a solution as concentrated as possible. It is known that PYP can be concentrated to 150 mg ml^−1^ or higher (M. Schmidt 2011, unpublished data), that is about 10 mmol l^−1^ or higher. In the following, we propose using a concentration of just one-fifth of this amount, namely about 2 mmol l^−1^. The current design specification of the LCLS is to produce an X-ray beam of perhaps 0.1 μm in diameter at the sample. There is some evidence that it is even possible to focus this beam further, to perhaps 0.01 μm or so (see SPring-8 website at www.spring8.or.jp/en/news_publications/research_highlights/no_50/). If the liquid jet containing the molecules under study can also be reduced to about 0.1 μm in width, then the volume of liquid illuminated by the XFEL beam will be about *π*5^2^.100 nm^3^. Then, the volume illuminated by the XFEL beam may be arranged to contain just a handful of molecules in random orientations and random positions along the beam. Each XFEL pulse is expected to contain about 10^12^ photons, and the electron cross section may be estimated from the Thomson radius *r*_0_ = 2.8 × (10*^−^*^15^) m to be about (8*π*/3) × (2.8 × 10*^−^*^15^)^2^ m^2^ = 0.656 × 10*^−^*^10^ nm^2^. Now, PYP contains some 1100 atoms or about 7000 electrons. Then, the cross section of scattering by a single molecule will be about 4600 × 10*^−^*^10^ nm^2^. If a single XFEL pulse is focused down to a spot diameter of 10 nm, then there will 10^12^ incident photons per 100 nm^2^, so the number of photons scattered by a single PYP molecule will be 4600 × 10*^−^*^10^ × 10^12^ × 10*^−^*^2^ = 4600 photons, sufficient for a reliable measurement of the angular pair correlations, when averaged over a large number of diffraction patterns, as proposed in this paper. Even in the outermost resolution ring (corresponding to a resolution of approx. 5 Å), the estimated number of photons per Shannon pixel (one may estimate some 120 such pixels from PYP at a resolution of about 5 Å) is about 0.5.

Owing to the expected coherence of an XFEL beam over a volume containing several particles, the scattering from multiple particles involves the addition of the *amplitudes* of the scattered X-rays, rather than only their intensities. However, as we see below, coherent interparticle interference effects are negligible if the average correlations from multiple diffraction patterns are calculated, each containing a *different* random distribution of identical particles.

First, we note that the average angular correlations from an ensemble of *N* particles per diffraction pattern may be written


6.1
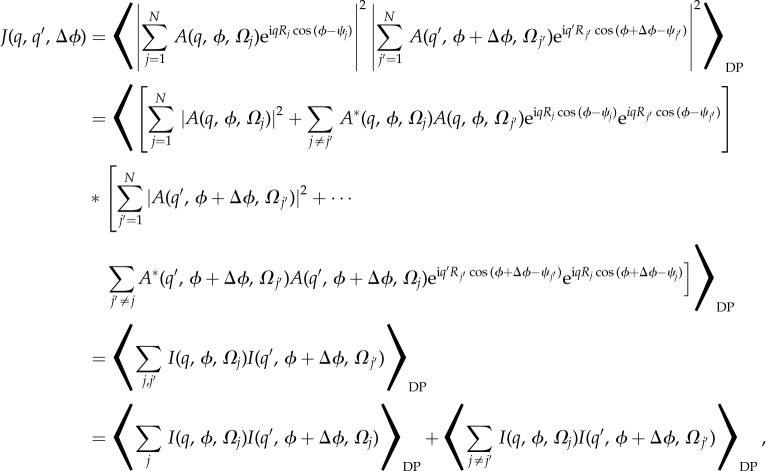


where *N* is the number of particles (of random orientations) contributing to a particular diffraction pattern, *Ω_j_* is the three-dimensional orientation of particle *j* (as usually specified by three Euler angles), and *R_j_* and *ψ_j_* are the two-dimensional polar coordinates of the positions of the *j*th particle in the plane perpendicular to the incident X-rays (this assumes that the scattering vector **q** is largely perpendicular to the X-ray beam). We took *R_j_* to be a random number between 0 Å and 200 Å, and *ψ_j_* a random number between 0 and 2*π*. (Of course, for uniformly distributed particles in real space, the probability of the higher *R_j_* values may be expected to be larger, and it may be necessary to take account of the finite thickness of the liquid jet, and the curvature of the Ewald sphere. We plan to test such more realistic models in future work, though we do not expect our main conclusions to be substantially different.)

**Figure 5.**
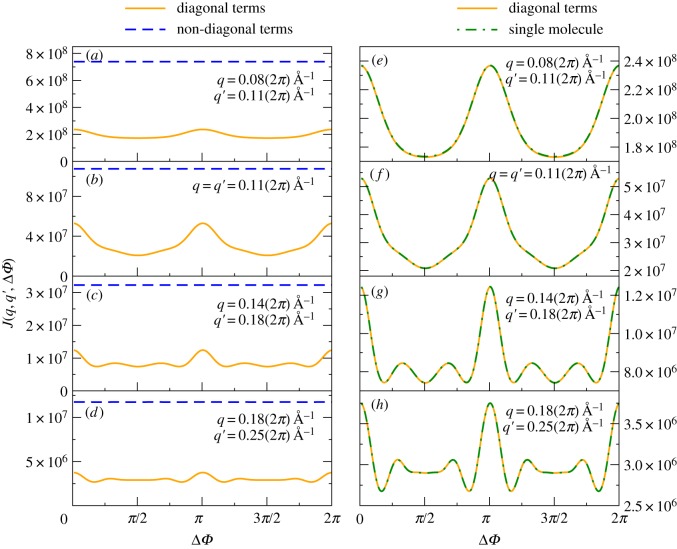
(*a*–*d*) Comparison of the ‘diagonal’ (solid line) and ‘off-diagonal’ terms (dashed line) in the intensity correlations (first and second terms after the last equality sign in (6.1)). These lines suggest although the ‘off-diagonal’ terms tend to dominate the measurable angular correlation functions, they tend to be of a relatively simple form (in this case, a constant as a function of angle Δ*ρ*, a fact that will probably allow their isolation, and hence removal. The remaining ‘diagonal’ terms contain the structural information of a single particle, as illustrated in (*e*–*h*). (*e*–*h*) Comparison of *J*(*q*, *q’*, Δ*ϕ*) curves obtained from a single particle per snapshot, and from five randomly oriented particles per snapshot (appropriately divided by 5). These calculations were performed for the small protein chignolin (PDB entry 1 UAO), but illustrate the general point that the sum of the diagonal terms in *J* (i.e. the intensity correlations from scattering by the same particles) are essentially independent of the number of such particles per snapshot (to within a *factor* of *N*, the number of particles per snapshot, *even if the particles are randomly oriented*). The radii *q* and *q’* of the resolution rings are chosen randomly from our range (up to a maximum value *q*_max_ = 2*π*/5 Å*^−^*^1^, or 5 Å resolution) to demonstrate that the result is essentially independent of the precise values of *q* and *q’*. (Online version in colour.)

A few words are in order about these equations. First, although the simulations assumed perfect coherence between X-rays scattered by different molecules, their random interparticle separations result in very small total contributions from the interference terms between different particles owing to the random phase terms arising from the random particle positions (the second and fourth terms after the second equality). The remaining sums over scattered *intensities* may be further subdivided into the case where the two correlated intensities arise from scattering by the *same* particle, and those where the two intensities arise from the scattering by *dissimilarly oriented* particles. Although the uncorrelated scattering is dominant, it tends to a constant, independent of angle (dashed lines on the left-hand panels of figure 5), a feature that will probably allow their isolation and removal from measured angular correlations (the proof of this will be the subject of a future paper), whereas the correlated scattering by the same particles add up to the same function of Δ*ϕ* (the solid curves in the left- and right-hand columns of the same figure) as expected for a single particle per snapshot, as illustrated in the right-hand column of figure 5. This should enable the same methods described in the first part of this paper to extract time-resolved structural changes of a single molecule even from experiments on solutions of randomly oriented molecules.

The right-hand column of figure 5 shows the variation of the quantities *J*(*q*, *q’*, Δ*ϕ*) from a set of five particles randomly oriented for any particular snapshot, but averaged over 10 000 diffraction patterns, with each particle in a random orientation in each. These correlations are plotted as a solid line. On these plots, we superimpose the same quantities calculated from diffraction patterns owing to single particles (hatched green curves). For ease of computation, these calculations were performed for an even smaller protein than PYP, namely chignolin (PDB entry 1 UAO). We stress that in both the case of a single particle per snapshot and five particles per snapshot, we averaged the correlations over 10 000 diffraction patterns. Thus, the correlations over the snapshots with five particles per snapshot involved averages over the orientations of some 50 000 particles, whereas those involving a single particle per snapshot involve an average over 10 000 randomly oriented particles. Almost perfect superposition is seen between these two sets of quantities, indicating the diagonal (scattering by same particle) correlations are essentially the same as those from scattering by a single particle. In a future paper, we will describe how to extract the diagonal terms from measured correlations consisting of a sum of diagonal and off-diagonal terms (the latter being angularly independent).

## 7. Discussion and conclusion

We propose as a method of analysis of the large number of XFEL diffraction patterns each measured from a small number of identical particles in random orientations the calculation of the average angular correlations of their intensities, because this quantity allows the extraction of a quadratic function *B_l_*(*q*, *q’*) of the spherical harmonic decomposition *I*_*lm*_(*q*) of intensities from a single particle at each resolution shell *q*. If the quantities *I*_*lm*_(*q*) may be extracted from this quadratic function, then this would enable the reconstruction of an *oversampled* three-dimensional diffraction volume of a single particle, and hence a deduction of the electron density of the particle. In the case of *ab initio* structure determination of a single particle, so far this has been demonstrated only for particles with a known high degree of symmetry, as for example the cases of icosahedral [21] or helical [22] viruses. Another circumstance in which structure solution is possible is when what is sought are small deviations from a known ‘dark’ structure of a protein in a time-resolved photoexcitation experiment. In this case, it is not necessary to assume *a priori* any symmetry of the difference structure. In an earlier paper, we demonstrated the structural information latent in the quantities *B_l_*(*q*, *q’*), which may be extracted by a systematic variation of the structural parameters, usually torsional angles, which by and large, specify the differences between the two sets of structures. The problem with this method is that if the residue or small number of residues undergoing the structural change on photoexcitation are unknown, the number of parameters which need to be optimized can be too large for even the best optimization methods available.

Our present simulations suggest that it is possible to find these structural differences at least to the accuracy of the difference Fourier methods of time-resolved crystallography without a prior knowledge of the part of the structure undergoing the structural change on photoexcitation. We do this by pointing out that for the small structural changes typical of fast photoexcitation experiments, the changes *δB_l_*(*q*, *q’*) in the quantities extractable from the photoexcitation experiments described, may be related *linearly* to the difference electron density sought. The linear relationship is specified by a matrix *M*, whose elements depend only on quantities calculable from a knowledge of the dark structure. What is more, the difference densities sought will be calculated in the frame of reference of the known dark structure, a fact that enables the difference electron density to be displayed on top of the known dark structure using widely used crystallographic display software such as Coot or Chimera (although Coot, designed for crystallography, tends to repeat the specified difference electron density periodically as if the structure were a crystal. Of course, in a crystal, the spatial relationship of atoms in different unit cells may be relevant, though it is not relevant in single particle work).

This means that the matrix *M* (and its inverse) can be calculated once and for all if a dark structure is known, and from this a difference electron density corresponding to each photoexcited structure investigated can be calculated by a simple matrix multiplication with the quantities *δB_l_*(*q*, *q’*) extractable from each experiment. By varying the time delays of each experiment, it should be possible to follow the fast time variation of the structures of photoexcited molecules in solution, rather than of crystallized proteins as in established methods of time-resolved crystallography. A full test on multiple particles of PYP per diffraction pattern will be reported on in a subsequent paper.

Scattering by the solvent would be expected from whatever parts vary rapidly. Subtracting scattered signals from solutions not containing the dissolved molecules would be expected to reduce the scattering from the solvent–air interface common to the solutions with and without the particles. However, there is another interface which exists only for the solutions containing the molecules, namely the solution–molecule interface, which will not be removed by this process. However, we may argue as do exponents of the technique of SAXS [23], that the effect of this interface is not so important at least at relatively low resolutions (below atomic resolution). At such resolutions, the solvent may be regarded as a uniform electron density *ρ_s_*, which we can take to be about 0.33 *e*^−^ Å*^−^*^3^. The difference from a solvent containing molecules and one without dissolved molecules is the existence of a volume occupied by the molecules that excludes the solvent. However, according to Babinet's principle, the scattering from such a solvent is equivalent to scattering by the volumes occupied by the particles of electron density −*ρ_s_*. Adding this quantity to the particle density (say *ρ_p_*(**r**)) results in an effective electron density of the particle of *ρ_p_*(**r**) − *ρ_s_* as is argued in the related technique of SAXS [23]. Thus, at least, at the relatively low resolution of our experiment, the effect of solvent scattering is merely to effectively reduce the electron density of the particle by *ρ_s_*, and so the modelling of the scattering is almost the same as that of particles *in vacuo*, except that the effective electron densities of the particles are reduced by *ρ_s_*.

If our results are confirmed in practice, then they suggest it may not be necessary to form crystals for following the fast time variations of the structure of photoexcited molecules. This is desirable for two reasons: (i) many proteins, particularly membrane proteins, are notoriously difficult to crystallize. Indeed, in protein structural work, crystallization is often the rate-limiting step. The freedom from the need to crystallize proteins may allow time-resolved structural changes of a larger class of proteins; (ii) proteins are generally not crystallized when they perform their functions in living entities in nature, but are rather suspended in aqueous solutions, just as in the present experiments. Consequently, there are no extra constraints owing to steric hindrance, which might exist in crystals, in the proposed experiments.

Of course, much still needs to be done to make this a routine method of structure determination. The effects of expected experimental noise need to be added [31] to the multiple simulated XFEL diffraction patterns to see if the same quantities can be recovered in the presence of such noise. And finally, the method still needs to be tested on experiments on known structures with XFEL radiation.

However, the initial indications of the possibility of recovery of information on fast structural changes of uncrystallized biomolecules in solution are exciting portends for the future.
